# Brain changes on magnetic resonance imaging in school-age children who had been preterm infants with intracranial hemorrhage

**DOI:** 10.1590/0100-3984.2016.0180

**Published:** 2017

**Authors:** Leandro Lopes Fernandes Alves, Marcia Salim de Martino, Cristina Ortiz Sobrinho, Adauto Dutra Moraes Barbosa

**Affiliations:** 1 MSc, Radiologist, Department of Maternal-Infant Care, Faculdade de Medicina da Universidade Federal Fluminense (UFF), Niterói, RJ, Brazil.; 2 MSc, Psychologist at the Outpatient Clinic for At-Risk Newborns of the Hospital Universitário Antônio Pedro, Universidade Federal Fluminense (UFF), Niterói, RJ, Brazil.; 3 PhD, Adjunct Professor of Pediatrics in the Department of Maternal-Infant Care, Faculdade de Medicina da Universidade Federal Fluminense (UFF), Niterói, RJ, Brazil.; 4 PhD, Full Professor of Pediatrics in the Department of Maternal-Infant Care, Faculdade de Medicina da Universidade Federal Fluminense (UFF), Niterói, RJ, Brazil.

**Keywords:** Newborn, Premature, Intracranial hemorrhage, Magnetic resonance imaging, Child, Unitermos:, Recém-nascido, Prematuro, Hemorragia intracraniana, Ressonância magnética, Criança

## Abstract

**Objective:**

To determine whether preterm infants diagnosed with intracranial hemorrhage (by transfontanellar ultrasound) at birth have cerebral lesions that are detectable by magnetic resonance imaging (MRI) upon reaching school age.

**Materials and Methods:**

MRI scans of the brain were obtained in 22 school-age children. Fifteen had presented intracranial hemorrhage at birth, and seven had not. We calculated the odds ratio (OR) for the occurrence of brain alterations detectable by MRI and the kappa index for discrepancies among the radiological reports.

**Results:**

The children without previous intracranial hemorrhage presented normal MRI findings. Of the 15 children with previous intracranial hemorrhage, 6 (40%) presented brain alterations on MRI: isolated ventricular alteration (dilation and asymmetry), in 2 (13.3%); and ventricular asymmetry accompanied by parenchymal lesion, in 4 (26.7%). The nine remaining children with previous intracranial hemorrhage (60%) presented normal MRI findings. The children with previous intracranial hemorrhage were more likely to present ventricular alteration (OR = 7.8) and parenchymal lesions (OR = 5.4).

**Conclusion:**

Ventricular and parenchymal brain alterations detected by MRI suggest isolated morphologic alterations that do not result in neurological impairment detectable on physical examination in school-age children.

## Abstract (pt)

**Objetivo:**

Investigar se ocorrem lesões cerebrais detectáveis na ressonância magnética (RM) cerebral em escolares que foram prematuros e tiveram hemorragia intracraniana diagnosticada pela ultrassonografia transfontanelar ao nascer.

**Materiais e Métodos:**

Foi realizada RM cerebral em 22 escolares, sendo 15 com história de hemorragia intracraniana e 7 não. Calculou-se a *odds ratio* (OR) de ocorrer alterações cerebrais detectáveis na RM cerebral e o valor de kappa para avaliar discrepâncias entre laudos radiológicos.

**Resultados:**

Os escolares sem hemorragia intracraniana apresentaram RM cerebral normal. Dos 15 pacientes com hemorragia intracraniana, 6 (40%) tiveram alteração na RM cerebral na idade escolar, sendo 2 (13,3%) com alteração ventricular isolada e 4 (26,7%) com assimetria ventricular associada a lesão parenquimatosa. Nove escolares (60%) com hemorragia intracraniana apresentaram RM cerebral normal. Os escolares com hemorragia intracraniana tiveram maior chance de apresentar alteração ventricular (OR = 7,8) e lesão parenquimatosa (OR = 5,4) na RM cerebral.

**Conclusão:**

As alterações ventriculares e parenquimatosas detectadas na RM cerebral sugerem alterações morfológicas isoladas, que não constituem comprometimento neurológico detectável ao exame físico na idade escolar.

## INTRODUCTION

Intracranial hemorrhage in the perinatal period is common among preterm infants, especially those with an extremely low birth weight (< 1000 g) or with a gestational age of less than 32 weeks^(1,2)^. It occurs in 20-25% of preterm infants born before 30 weeks of gestation or with a birth weight of less than 1500 g. The long-term outcomes of infantile intracranial hemorrhage, especially during childhood, remain a matter of debate^(3)^, and there have been no brain imaging studies of this problem.

The objective of this study was to use magnetic resonance imaging (MRI) to identify brain lesions in school-aged children who had been preterm infants with some degree of intracranial hemorrhage at birth.

## MATERIALS AND METHODS

This was a cross-sectional, descriptive, analytical case-control study, carried out at Hospital Universitário Antônio Pedro (HUAP), in the city of Niterói, Brazil, between July 2014 and June 2015. This study respected all of the terms that govern Brazilian National Health Council Resolution no. 196/96 on ethics in research involving human beings and was approved by the Human Research Ethics Committee of the Fluminense Federal University School of Medicine. We included school-age children who had been born prematurely in a hospital maternity ward between January 2006 and December 2008 and had presented intracranial hemorrhage, diagnosed by transfontanellar ultrasound (TFUS), by day 7 of life. All of those children were being followed at a hospital outpatient clinic and were collectively designated the intracranial hemorrhage group. We also included, as a control group, children who were born prematurely in the aforementioned period but presented with no signs of hemorrhage on TFUS at birth. Children whose medical records did not contain TFUS data or contained incomplete or discordant data were excluded, as were those who had presented any neurological disorder, unrelated to intracranial hemorrhage, during childhood and those who declined to undergo MRI of the brain (because of a fear of the procedure) or in whom the examination was of poor technical quality due to movement artifacts.

Data were collected through an active search of the records of the HUAP Outpatient Clinic for At-Risk Newborns and were placed on a list that included the name, date of birth, mother’s name, and medical chart number of each subject. After a careful analysis of the patient charts, we evaluated the following variables: maternal complications during pregnancy; type of delivery; gestational age; birth weight; 1-min and 5-min Apgar score; length of hospital stay; and complications in the neonate in the postnatal period, including sepsis, the need for (and duration of) mechanical ventilation, intracranial hemorrhage, jaundice, and hyaline-membrane disease.

The TFUS examinations evaluated in the study were described in the patient charts, with characterization of the lesion by its location, appearance on imaging, and degree of intracranial hemorrhage. All TFUS examinations were performed with the same ultrasound system (Nemio; Toshiba Medical Systems, Tokyo, Japan), with convex and linear transducers (6-10 MHz). Some of the examinations included photographs (printed on ultrasound-specific paper) taken at the time of the test.

The MRI scans of the brain were performed after the parents or legal guardians of the children had been invited to participate and had given written informed consent for the minors to participate in the study. The scans were obtained in a 1.5 T scanner (Signa HDxt; General Electric Medical Systems, Waukesha, WI, USA), with a dedicated head coil. The following anatomical and functional sequences were obtained: axial T1-weighted, T2-weighted, fluid-attenuated inversion recovery (FLAIR), and susceptibility-weighted sequences; sagittal T1-weighted sequences; and coronal T2-weighted sequences. All procedures followed established protocols for performing MRI of the brain in pediatric patients^(4-6)^.

For MRI of the brain, no gadolinium or sedation was used. The MRI brain scans were uploaded to a computerized server and evaluated at different times by two radiologists from the HUAP Radiology Department who did not participate in the study and were blinded to the perinatal TFUS data. The MRI reports were descriptive, including an evaluation of the cerebral parenchyma and ventricular system. The examinations that showed alterations were separated by the location of the alteration (cerebral parenchyma or ventricular system) and by the type of lesion (ventricular asymmetry, ventricular dilatation, gliosis, or hemoglobin residue).

The radiologists who described the TFUS and the MRI reports were professionals who held the title of specialist awarded by the Brazilian College of Radiology and Diagnostic Imaging, were university professors, and had at least 15 years of experience in the specialty.

All data were tabulated in the Excel program, and the statistical analysis was performed with the Statistical Package for the Social Sciences, version 18.0 (SPSS Inc., Chicago, IL, USA). Initially, the Kolmogorov-Smirnov test was used in order to evaluate the distribution of the data collected. Using bivariate analysis, we evaluated quantitative variables with the Student’s *t*-test and qualitative variables with the chi-square test. For infants who presented with intracranial hemorrhage, we calculated odds ratios (ORs) in order to quantify the risk of the occurrence of changes in brain structure that would be detectable by MRI. The kappa index was calculated in order to evaluate discrepancies between the aspects observed by the two radiologists.

## RESULTS

In our initial search of patient charts, we identified 80 potentially eligible children-65 who had been diagnosed with intracranial hemorrhage at birth and 15 who had not. We were able to contact and obtain consent for the MRI of the brain from the parents or legal guardians of 32 (40%) of those 80 children. Of those, 10 were unable to perform the test for any of a variety of reasons. Therefore, the final sample comprised 22 children, of whom 15 (68.2%) had been diagnosed with intracranial hemorrhage in the perinatal period and seven (31.8%) had not.

At the time of the MRI of the brain, the ages of the children included in the study ranged from 6 years and 5 months to 8 years and 6 months, the mean age being 7 years ± 7 months and the median age being 6 years and 9 months. In terms of the age at which the MRI was performed, there was no significant difference between the two groups (Table 1).

**Table 1:** Quantitative variables at birth in the groups of children studied.

	Without intracranial hemorrhage ( *n* = 7)			With intracranial hemorrhage (*n* = 15)		*P*
	Mean ± standard deviation 95% CI			Mean ± standard deviation 95% CI		
Gestational age (weeks)	31.86 ± 1.9	30.1–33.6		31.5 ± 4.6	30.6–32.4	0.624
Birth weight (g)	1398.86 ± 373.6	1053.3–1744.4		1408.3 ± 309.4	1237.0–1579.7	0.950
1-min Apgar score	6.71 ± 1.5	5.3–8.1		6.1 ± 2.7	4.6–7.6	0.585
5-min Apgar score	8.29 ± 0.9	7.4–9.2		8.00 ± 1.9	6.9–9.1	0.717
Hospital stay (days)	35.14 ± 15.5	20.8–49.4		38.9 ± 22.9	26.2–51.6	0.361
Age at brain MRI (years)	7.3 ± 0.5	6.6–8.0		7.0 ± 0.6	6.6–7.3	0.324

95% CI, 95% confidence interval.

**Figure 1:**
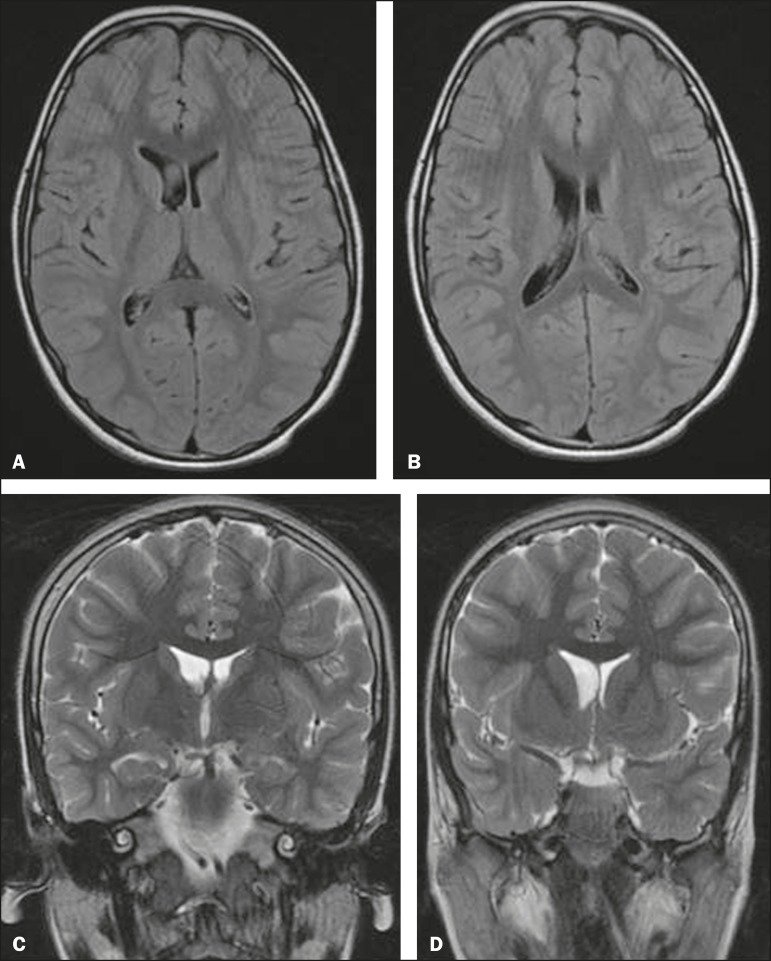
MRI of the brain showing ventricular asymmetry (right lateral ventricle greater than the left) without parenchymal alteration. Axial FLAIR sequence (A,B) and coronal T2-weighted sequence (C,D), both without gadolinium or sedation. On the perinatal TFUS, this subject had presented grade I intracranial hemorrhage (subependymal hemorrhage).

Among the 22 children in the sample as a whole, the MRI findings were normal in 16 (72.8%) and revealed some type of alteration in 6 (27.2%): isolated ventricular alterations (Figure 1) in 2 (9%); and concomitant ventricular and parenchymal alterations in 4 (18%). When the 15 children who had a history of intracranial hemorrhage were analyzed separately, those proportions increased-6 (40%) presented with some type of brain alteration on MRI: 2 (13.3%) with isolated ventricular alterations; and 4 (26.7%) with concomitant ventricular and parenchymal alterations. None of the children in our study sample presented parenchymal alteration without ventricular alteration. None of the seven children in the perinatal intracranial hemorrhage group presented detectable alterations on the MRI of the brain.

**Figure 2:**
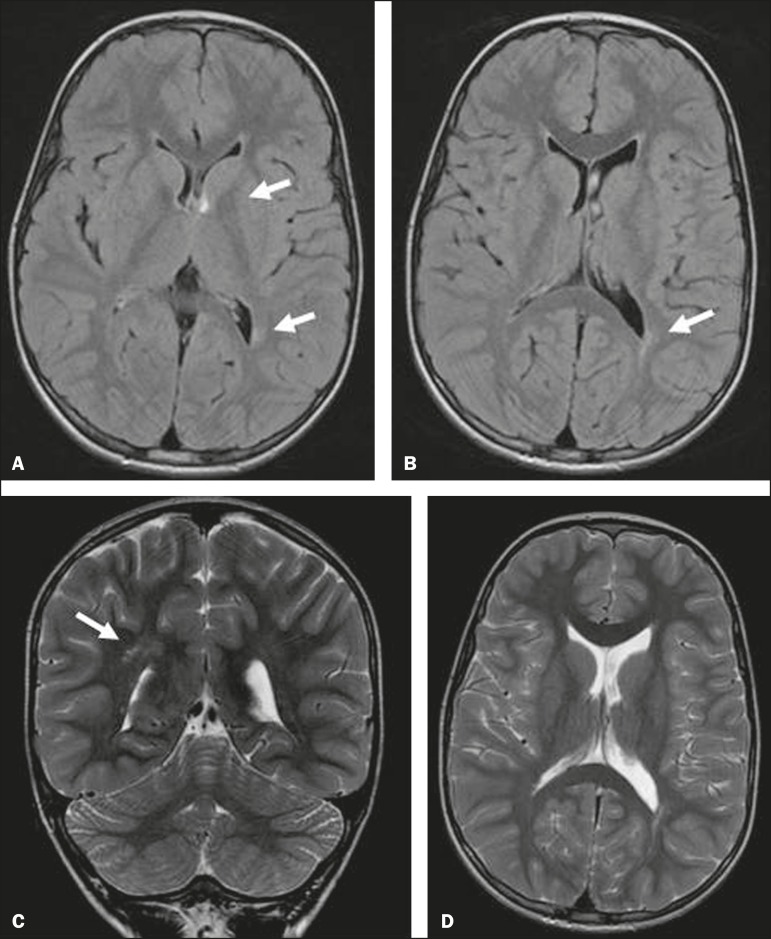
MRI of the brain showing ventricular asymmetry (left lateral ventricle greater than right) and periventricular foci of gliosis (arrows). Axial FLAIR sequence (A,B), coronal T2-weighted sequence (C), and axial T2-weighted sequence (D), all without gadolinium or sedation. On the perinatal TFUS, the subject had presented grade II intracranial hemorrhage (subependymal hemorrhage accompanied by intraventricular clots).

**Figure 3:**
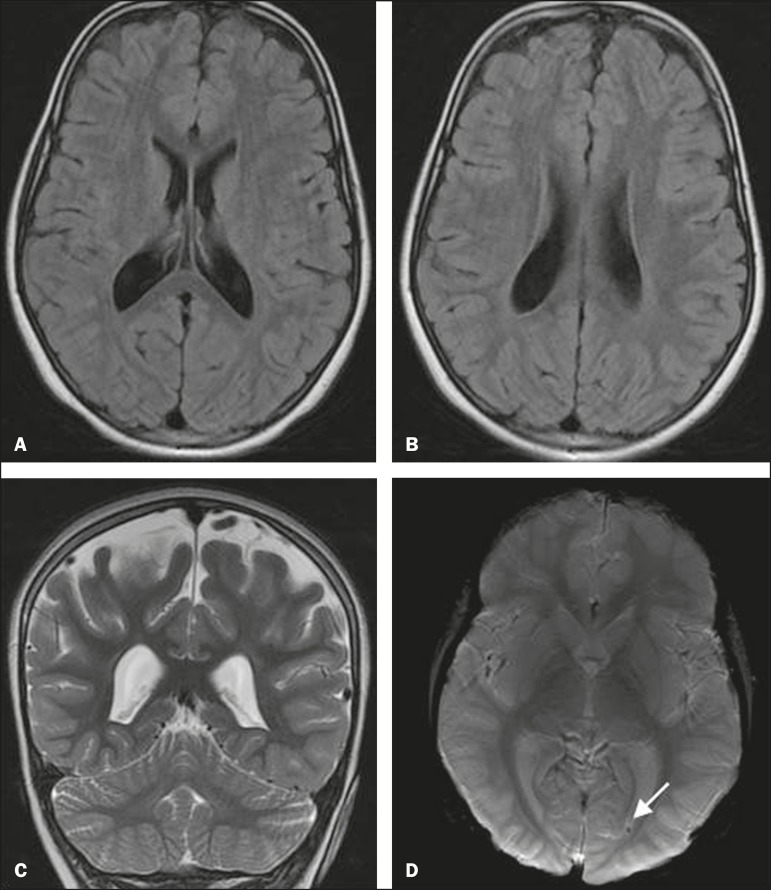
MRI of the brain showing symmetric ventricular dilatation and periventricular focus of hemoglobin degradation (arrow). Axial FLAIR sequence (A,B), coronal T2-weighted sequence (C), and axial susceptibility-weighted sequence (D), all without gadolinium or sedation. On the perinatal TFUS, the subject had presented grade III intracranial hemorrhage.

Of the four children with parenchymal abnormalities, three had periventricular foci of gliosis (Figure 2) and one had periventricular foci of residues of hemoglobin degradation (Figure 3). Of the six children with ventricular changes (including the four with concomitant ventricular and parenchymal alterations), five presented ventricular asymmetry and one presented symmetric dilation of the lateral ventricles.

In comparison with the children in the control group, those in the perinatal intracranial hemorrhage group were more likely to show a change in the size of the cerebral ventricle (OR = 7.8; 95% confidence interval = 0.38-15.0; *p* = 0.18), as well as being more likely to present parenchymal lesions (OR = 5.4; 95% confidence interval = 0.25-125.58; *p* = 0.28).

The kappa index for the concordance between the MRI reports provided by the two radiologists was 0.741, showing substantial agreement between them (*p* = 0.001).

## DISCUSSION

Most of the studies of the topic in question have addressed the imaging follow-up of children who presented intracranial hemorrhage only during postnatal hospitalization, such as that conducted by Dyet et al.^(7)^, or have addressed neuropsychological and behavioral aspects (without imaging studies) in children or adolescents, such as that conducted by Luu et al.^(8)^, who evaluated the behavior of subjects at 12 years of age. There are no clear correlations among postnatal survival, the degree of intracranial hemorrhage/postnatal injury, and the brain lesions observed during childhood.

Among the brain MRI findings in the children evaluated in the present study, ventricular dilation accompanied by periventricular foci of hemoglobin degradation, as a result of grade III intracranial hemorrhage in the perinatal period, was observed in one child. In that child, the perinatal TFUS examination had shown intracranial hemorrhage accompanied by ventricular dilatation, although not extending to the adjacent cerebral parenchyma, which explains the ventricular dilation seen on the MRI of the brain. The foci of residual periventricular bleeding seen on the MRI scans could be explained either by the lower sensitivity of the TFUS in comparison with MRI, as demonstrated by Childs et al.^(9)^, or by small areas of periventricular venous infarction, as previously described here. The remaining children who showed parenchymal changes on the MRI of brain had presented grade I or II intracranial hemorrhage on the perinatal TFUS and presented discrete foci of periventricular gliosis on the MRI, which was unexpected, given that there have been no previous reports of intracranial hemorrhage extending to the parenchyma. These findings could be related to the possibility that discrete ischemic changes to the white matter are visible on MRI and not on TFUS^(10,11)^. A study conducted by Debillon et al.^(12)^ showed that, although TFUS is quite efficient in detecting severe white matter lesions, MRI of the brain is necessary for the diagnosis of mild lesions of the white matter.

One finding that caught our attention was the number of children in the perinatal intracranial hemorrhage group who presented ventricular asymmetry on MRI of the brain. This finding, which is often routinely described as a constitutional change, did not occur in our control group subjects, nor has it been reported in the literature, making it a matter worthy of further investigation. Ventricular asymmetry, when found in examinations performed for other causes, such as computed tomography of the brain, should motivate the pediatrician to conduct a more careful investigation using MRI, especially for small foci of gliosis and residual bleeding^(13)^, which, when present, can indicate a pathological history of intracranial hemorrhage at birth.

In the present study, a large number of subjects who presented with intracranial hemorrhage on perinatal TFUS showed no detectable changes on MRI of the brain. This was expected in those who had presented milder (grade I or II) intracranial hemorrhage, which, as previously mentioned, does not typically result in sequelae. However, the children who had presented grade III or IV intracranial hemorrhage also had normal MRI results. This could be explained by adaptive mechanisms of the developing brain and should serve to stimulate research on this theme, as suggested by Ment et al.^(14)^.

## CONCLUSION

The findings of ventricular dilatation/asymmetry and parenchymal lesion suggest isolated morphological alterations that, despite being observed in childhood in the subjects who had presented intracranial hemorrhage in the perinatal period, are not detectable signs of neurological impairment, although they are, respectively, 7.8 and 5.4 times more likely to be found on the MRI brain scans of subjects who presented intracranial hemorrhage at birth than on those of subjects who did not.
